# Identification of a Kavain Analog with Efficient Anti-inflammatory Effects

**DOI:** 10.1038/s41598-019-49383-8

**Published:** 2019-09-10

**Authors:** Olivier Huck, Xiaxian Han, Hannah Mulhall, Iryna Gumenchuk, Bin Cai, James Panek, Radha Iyer, Salomon Amar

**Affiliations:** 1 Université de Strasbourg, Faculté de Chirurgie-Dentaire, 8 rue Sainte-Elisabeth, 67000 Strasbourg, France; 2 INSERM (French National Institute of Health and Medical Research), UMR 1260, Regenerative Nanomedicine, Fédération de Médecine Translationnelle de Strasbourg (FMTS), Strasbourg, France; 3 Departments of Pharmacology, Microbiology and Immunology, New York Medical College, Valhalla, NY 10595 NY USA; 4 Department of Chemistry, Boston University, Boston, MA USA

**Keywords:** Pharmacology, Bacterial infection

## Abstract

Kavain, a compound derived from *Piper methysticum*, has demonstrated anti-inflammatory properties. To optimize its drug properties, identification and development of new kavain-derived compounds was undertaken. A focused library of analogs was synthesized and their effects on *Porphyromonas gingivalis* (*P. gingivalis)* elicited inflammation were evaluated *in vitro* and *in vivo*. The library contained cyclohexenones (5,5-dimethyl substituted cyclohexenones) substituted with a benzoate derivative at the 3-position of the cyclohexanone. The most promising analog identifed was a methylated derivative of kavain, Kava-205Me (5,5-dimethyl-3-oxocyclohex-1-en-1-yl 4-methylbenzoate.) In an *in vitro* assay of anti-inflammatory effects, murine macrophages (BMM) and THP-1 cells were infected with *P. gingivalis* (MOI = 20:1) and a panel of cytokines were measured. Both cell types treated with Kava-205Me (10 to 200 μg/ml) showed significantly and dose-dependently reduced TNF-α secretion induced by *P. gingivalis*. In BMM, Kava-205Me also reduced secretion of other cytokines involved in the early phase of inflammation, including IL-12, eotaxin, RANTES, IL-10 and interferon-γ (*p* < 0.05). *In vivo*, in an acute model of *P. gingivalis*-induced calvarial destruction, administration of Kava-205Me significantly improved the rate of healing associated with reduced soft tissue inflammation and osteoclast activation. In an infective arthritis murine model induced by injection of collagen-antibody (ArthriomAb) + *P. gingivalis*, administration of Kava-205Me was able to reduce efficiently paw swelling and joint destruction. These results highlight the strong anti-inflammatory properties of Kava-205Me and strengthen the interest of testing such compounds in the management of *P. gingivalis* elicited inflammation, especially in the management of periodontitis.

## Introduction

Periodontitis is an inflammatory disease leading to the destruction of tooth-supporting tissues, that may be induced by dysbiosis^1^. Over the last decades, several links between periodontitis and systemic diseases have been suggested^2^. Periodontitis has been associated with increased incidences of adverse cardiovascular events, adverse pregnancy outcomes, diabetes, and rheumatoid arthritis^3–6^. Bacterial spreading from diseased periodontal sites through the bloodstream has been suggested as one of the major mechanisms involved, since periodontal pathogens have been detected in several distant tissues and organs such as blood vessels^7^ and joints^8^. The detrimental role played by the major periodontal pathogen *Porphyromonas gingivalis* (*P. gingivalis*), a keystone pathogen implicated in periodontal dysbiosis^1,9^ has been extensively studied. For example, *P. gingivalis* is able to invade host immune cells and to hijack host immune responses, leading to exacerbated inflammation and tissue destruction, both locally and at a distance^10,11^.

Kavain, a compound extracted from the *Piper methysticum* plant, has recently been described as a promising anti-inflammatory agent and has been evaluated in several *in vitro* and animal models^12–15^. In macrophages stimulated by *Escherichia coli* lipopolysaccharide (LPS), treatment with kavain significantly reduced TNF-α secretion,^13^ while in another cellular model, kavain was shown to inhibit RANKL-induced osteoclast formation^15^. In an attempt to optimize kavain’s anti-inflammatory effects and to reduce potential side effects, an effort has been made to identify kavain analogs^16^. One aspect of this effort to synthesize and validate new kavain-analog candidates, has been a focus on the need to reduce the susceptibility of kavain to enzymatic degradation associated with the presence of an α-, β-unsaturated lactone moiety. One resulting compound, Kava-241, is a synthesized kavain analog that has already demonstrated anti-TNF-α properties and the ability to reduce both *P. gingivalis*-induced periodontitis and arthritis^17,18^. In macrophages infected with *P. gingivalis*, the TNF-α decrease observed after treatment with this compound was associated with a reduced activation of Toll-like receptor (TLR)-related pathways and lipopolysaccharide-induced TNF factor (LITAF)^18^.

The present study aimed to identify additional new kavain-derived analogs and to evaluate their anti-inflammatory properties *in vitro* and *in vivo*. The most promising analog identified was Kava-205Me, a methylated kavain analog.

## Materials and Methods

### Synthesis of kavain analogs

**Figure 1:**
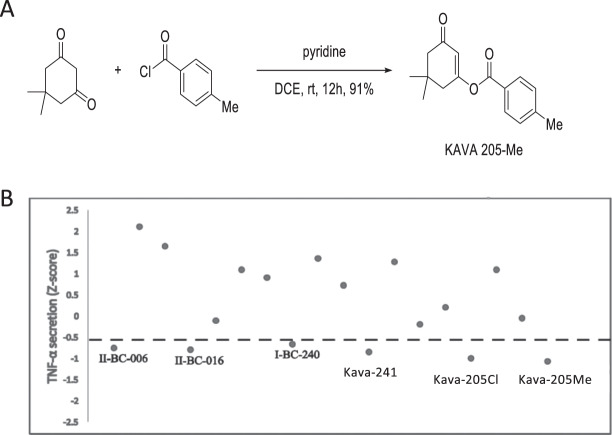
(**A**) The synthesis of Kava-205Me is based on the O-acylation of the highly enolizable cyclic 1,3-diketones. Accordingly, treatment of 1,2-dichloroethane solution of commercially available 1,3-cyclohexanedione with 4-methylbenzoyl chloride in the presence of pyridine efficiently provided the O-acylated enol derivative Kava-205Me. (**B**) Kava-205 chlorinated and methylated forms reduce TNF-α in BMM infected with *P. gingivalis*. Kavain derived compounds identified through SAR were tested (40 μg/ml). Compounds were plotted according to z-score of TNF-α inhibition. Chemical formula and structure of others tested analogs are presented in Supplementary Fig. 1.

A useful approach to optimize potential lead compounds is commonly referred to as a structure–activity-relationship (SAR) study. Specifically, efforts are directed toward identifying the correlations between substructures of compounds and their biological properties. A series of chemical modifications on the specific sites of the lead compounds are made to improve the potency and pharmaceutical properties. In our previous SAR study^16^, we found that one ring-opened analogue bearing an α,β-unsaturated ester displayed ≥50% suppression of TNF-α secretion. In addition, medicinal chemistry studies have revealed the effect of a methyl group in promoting and enhancing compound potency^19,20^. In light of these findings, we generated a focused library of cyclohexenones (5,5-dimethyl substituted cyclohexenones) substituted with a benzoate derivative at the 3-position of the cyclohexanone, which included the analog Kava-205Me (Fig. 1A). Kava-205Me was tested *in vitro* at the concentration ranging from 10, 50, 200 μg/ml.

### Porphyromonas gingivalis culture

The *Porphyromonas gingivalis* 381 strain was cultured and maintained in brain-heart infusion media supplemented with hemin (5 μg/mL, Sigma-Aldrich, St. Louis, MO), and menadione (1 μg/mL, Sigma-Aldrich, St. Louis, MO) in an anaerobic environment (AnaeroPack-Anaero, Mitsubishi Gas Chemical Co.; New York, NY) as previously described^10^.

### Mouse bone marrow macrophage (BMM) isolation and infection

BMM were isolated from mouse bone marrow as previously described^10^. Briefly, after euthanasia, femurs and tibias were harvested and the bone marrow was flushed from the medullar cavity with collection media (DMEM, 10% FBS, and 1% penicillin-streptomycin). Cells were cultured in 30% L-929 conditioned RPMI media at a density of 10^5^ cells/mL. L-929 (ATCC no. CCL-1) is a murine fibroblast cell line that is used as a source of macrophage colony-stimulating factor (M-CSF)^21^. After one week, cells had differentiated into BMM. The day of infection, cells were seeded in a 24-wells plate and after PBS wash, *P. gingivalis* 381 was added for 4 h to the BMM cultures at a MOI = 20:1^22^.

### THP-1 cell culture

THP-1 (ATCC® TIB-202™) cells were grown in RPMI medium containing 1% penicillin/streptomycin, 10% fetal bovine serum and β-mercaptoethanol (0.05 mM) in 5% CO_2_ at 37°. Infection was performed as described for BMM.

### TNF-α ELISA

The supernatants from infected cells and mouse serum were evaluated by ELISA for the detection of TNF-α concentration with an Invitrogen kit (KMC3011, ThermoFisher, Dublin, OH, USA). ELISA immunoreactivity was quantified using a microplate reader (Bio-Rad, Hercules, CA, USA).

### Bioplex pro Mouse cytokine 23-plex assay

A cytokine 23-plex kit (BioRad, CA, USA, Cat #M60009RDPD) was used according to manufacturer’s instructions to measure the concentrations of eotaxin, G-CSF, GM-CSF, IFN-γ, IL-1α, IL-1β, IL-2, IL-3, IL-4, IL-5, IL-6, IL-9, IL-10, IL-12 (p40), IL-12 (p70), IL-13, IL-17A, KC MCP-1 (MCAF), MIP-1α, MIP-1β, RANTES, and TNF-α in supernatants from BMM. The fluorescent signal intensity was measured using Bioplex 200 system. Cytokine concentrations were determined using standard curves generated using Bioplex manager software (V4.1).

### RNA extraction and quantitative real-time PCR (qRT-PCR)

Total RNA from BMM was isolated and purified with a Rneasy Mini kit according to the manufacturer’s instructions (Qiagen, Hilden, DE, USA). cDNA from total RNA was synthesized (50–100 ng RNA/20 μl) using a QuantiTect Reverse transcription kit according to the manufacturer’s instructions (Qiagen). qRT-PCR was performed using the Taqman Fast Advanced Master Mix (Applied Biosystems, Foster City, CA, USA) and was run for the following gene using the probe provided by Thermofisher: acid phosphatase 5 (ACP5) (Mn00475698_m1). qRT-PCR assays were performed in duplicates on an Applied Biosystems QuantStudio 5 Real-Time PCR system. The data were analyzed using QuantStudio 5 software V1.4. The gene expression levels were normalized to β-actin for BMM samples respectively and expressed relative to unstimulated controls following the 2-ΔΔCT method.

### Mouse calvarial bone resorption model

The 8- to 12-week-old wild-type C57BL/6 J mice used in this study were purchased from Taconic Laboratories (Germantown, NY). All mice were housed 3/cage at the New York Medical College Animal Facility, on a 12 h light dark cycle and *ad libitum* access to food and water. All animals were cared by veterinary staff for house boundary, biological analysis and maintenance. All procedures were approved by the NYMC IACUC committee and follow ARRIVE guidelines. The mice were randomly allocated into the following three groups (5 mice/group): (i) PBS (ii) *P. gingivalis* (iii) *P. gingivalis* + Kava-205Me concurrently. Mice were anesthetized by intraperitoneal injection of ketamine-xylazine. The heads of mice were shaved, and then live bacteria resuspended in 100 μl of PBS were injected subcutaneously with a 30.5-gauge needle at a point on the midline of the skull between the ears and eyes, as we have described previously^10^. The dose of *P. gingivalis* (5 × 10^8^) was injected. In the treatment group, 1 mg of Kava-205Me was concurrently injected. Mice were euthanized 4 days post injection. The size of the lesion (area in square millimeters) resulting from the injection in each animal was analyzed using ImageJ software.

### Induction of arthritis and scoring

Six-weeks-old, pathogen-free DBA1/BO male mice were obtained from Taconic Laboratories. Arthritis was induced by two consecutive intraperitoneal injections of ArthritoMab (AB) antibody cocktail (Chondrex, WA, USA). 3.5 mg were injected at baseline and a second injection of 1 mg was done at day 4. A sample of 5 mice/group was considered based on our previous data^18^ and all animals were randomly allocated in each group. For *P. gingivalis* injected groups, 3 intraperitoneal injections of 5 × 10^8^ bacteria/100 μl were administered. Mice were euthanized after 35 days by CO_2_ inhalation. All four paws were evaluated by a reviewer blinded to the treatment group to score arthritis using a visual qualitative assessment scoring as follows: (0) no paw swelling, (1) mild swelling, (2) moderate swelling, (3) severe swelling.

### Injection of *P. gingivalis* and Kava-205Me compound

In Kava-205Me treated groups, 8 intraperitoneal injections (40 mg/kg) were administered. The first injection was performed 3 days after the first AB injection (day 0) and at days 5, 7, 10, 13, 14, 18. As a control, same volume of the DMSO used to dissolve Kava-205 powder was used.

### Tissue preparation

Phalangeal joints and intact surrounding tissues were fixed with 4% freshly prepared paraformaldehyde (Sigma-Aldrich, St. Louis, MO) in PBS (pH 7.2) for 24 h at 4 °C. Following fixation, specimens were consecutively washed with 5%, 10%, and 15% glycerol (American Bioanalytical, Natick, MA) in PBS, each for 15 min at 4 °C and decalcified in an EDTA solution (Sigma-Aldrich, St. Louis, MO) for 14 days at 4 °C. Samples were then immersed in 30% sucrose (Sigma-Aldrich, St. Louis, MO) in PBS until embedding. Tissue blocks were embedded with a HISTOPREP^®^ compound (Fisher Scientific, Hanover Park, IL) for cryostat sectioning. Serial mesiodistal sections (5 μm) parallel to the long axis of the phalangeal joint were made and stained with hematoxylin (Fisher Scientific, Pit, IL) – eosin (ACROS Organics, Morris Plains, NJ).

### TRAP staining

Osteoclasts were detected by TRAP staining. 5 µm thick histological slides were exposed to the TRAP solution containing N,N-dimethylformamide (EM Science), 3.7 mM of fast red violet LB di-azonium salt (Sigma), 6.4 mM of tartaric acid (Sigma), and 0.4% MgCl_2_ in 0.2 M sodium acetate buffer (pH 5.0) for 10 minutes at 37 °C. The slides were then washed for 30 minutes before being counter-stained with hematoxylin for 5 seconds. Osteoclasts were identified as being positively stained for TRAP and possessing a ruffled border with an underlying lacuna.

### Histological scoring

Samples were scored for inflammation, bone destruction, bone formation/repair and for cartilage destruction. To score inflammation, a 0–4 scale was used with 0 corresponding to no signs of inflammation, 1 to mild infiltration of inflammatory cells, 2 to mild inflammation with small hyperplasia in the synovial lining layer, 3 to synovial edema, hyperplasia and more pronounced inflammation, and 4 to severe synovial hyperplasia and cellular infiltration. Bone destruction was scored using a 0 to 4 scale with 0 corresponding to no signs of bone destruction, 1 to osteoclast activation, 2 to presence of some osteoclast lacunae, 3 to presence of many osteoclast lacunae and signs of bone resorption, and 4 to severe bone resorption and erosion.

### Bone histomorphometry

For each animal, two slides, each containing three tissue sections with the largest number of bone marrow cells (six specimens total), were analyzed. For each tissue section, the microscopic fields with the most resorption were studied. The osteoclast index, which represents the number of osteoclasts per millimeter of trabecular bone surface, was measured.

The percentage of bone surface covered by osteoclasts was also quantified. This was calculated as the sum of the lengths of the osteoclasts containing lacunae (active eroded area) divided by the total trabecular bone perimeter.

### Statistical analysis

All experiments have been performed at least in triplicate. Data were analyzed for statistical significance with XLStat (Addinsoft, New York, NY, USA). *P* values were calculated with the Mann-Whitney U-test or ANOVA one-way t-test for multiple comparisons. Results were considered significant at *p ≤ 0.05, **p ≤ 0.01, ***p ≤ 0.001. Data are presented as mean ± standard error of the mean (SEM).

## Results

### Screening of selected kavain-derived analogs leads to identification of Kava-205Me

**Figure 2:**
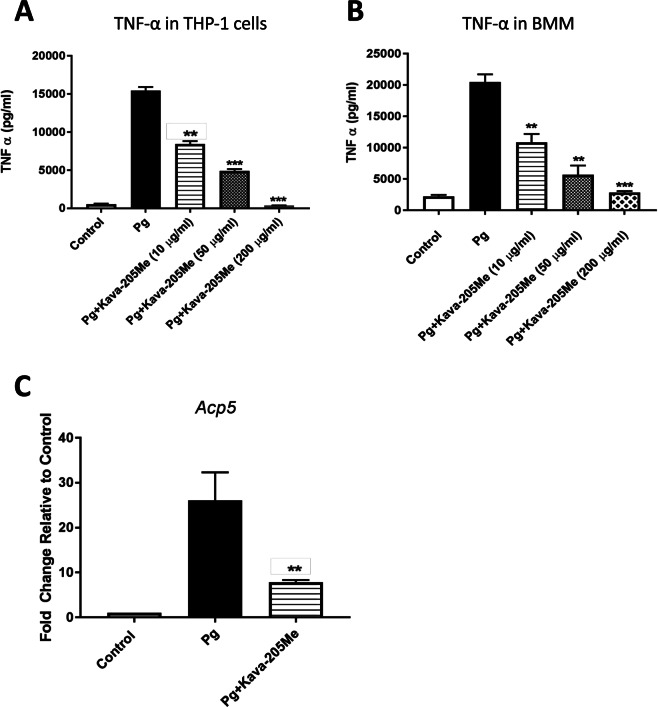
Kava-205Me (10, 50, 200 μg/ml) reduced significantly TNF-α secretion induced by *P. gingivalis* in supernatants of human THP1 cells **(A)** and in BMM **(B)** in a dose-dependent manner. Kava-205Me (50 μg/ml) reduced significantly ACP5 gene expression in BMM infected with *P. gingivalis* **(C)**. **p < 0.01; ***p < 0.001.

To identify compounds with strong anti-inflammatory properties, kavain analogs were tested in a screening assay based on TNF-α inhibition in BMM infected with *P. gingivalis* (Fig. 1B). The chemical structure of all tested compounds is described in Fig. 1 Supplemental Information. This screen identified Kava-205Cl (5,5-dimethyl-3-oxocyclohex-1-en-1-yl 4-chlorobenzoate) and its methylated analog Kava-205Me (5,5-dimethyl-3-oxocyclohex-1-en-1-yl 4-methylbenzoate) (Fig. 1B) as effective inhibitors of TNF-α secretion (z = −0.99 and z = −1.07 respectively). In THP-1 and BMM infected with *P. gingivalis*, a dose-response evaluation was performed and showed that treatment with Kava-205Me reduced significantly TNF-α secretion even at the lowest tested dose (10 μg/ml) (Fig. 2A,B) emphasizing the anti-inflammatory effect of tested compound in both murine and human cells. Moreover, no significant cytotoxicity was observed when using Kava-205Me at 10 and 50 μg/ml concentrations *in vitro* (Supplementary Fig. 2). Therefore, the 50 μg/ml concentration was selected for further experiments.

### Kava-205Me significantly reduced *P. gingivalis*-induced cytokine secretion from macrophages

**Figure 3:**
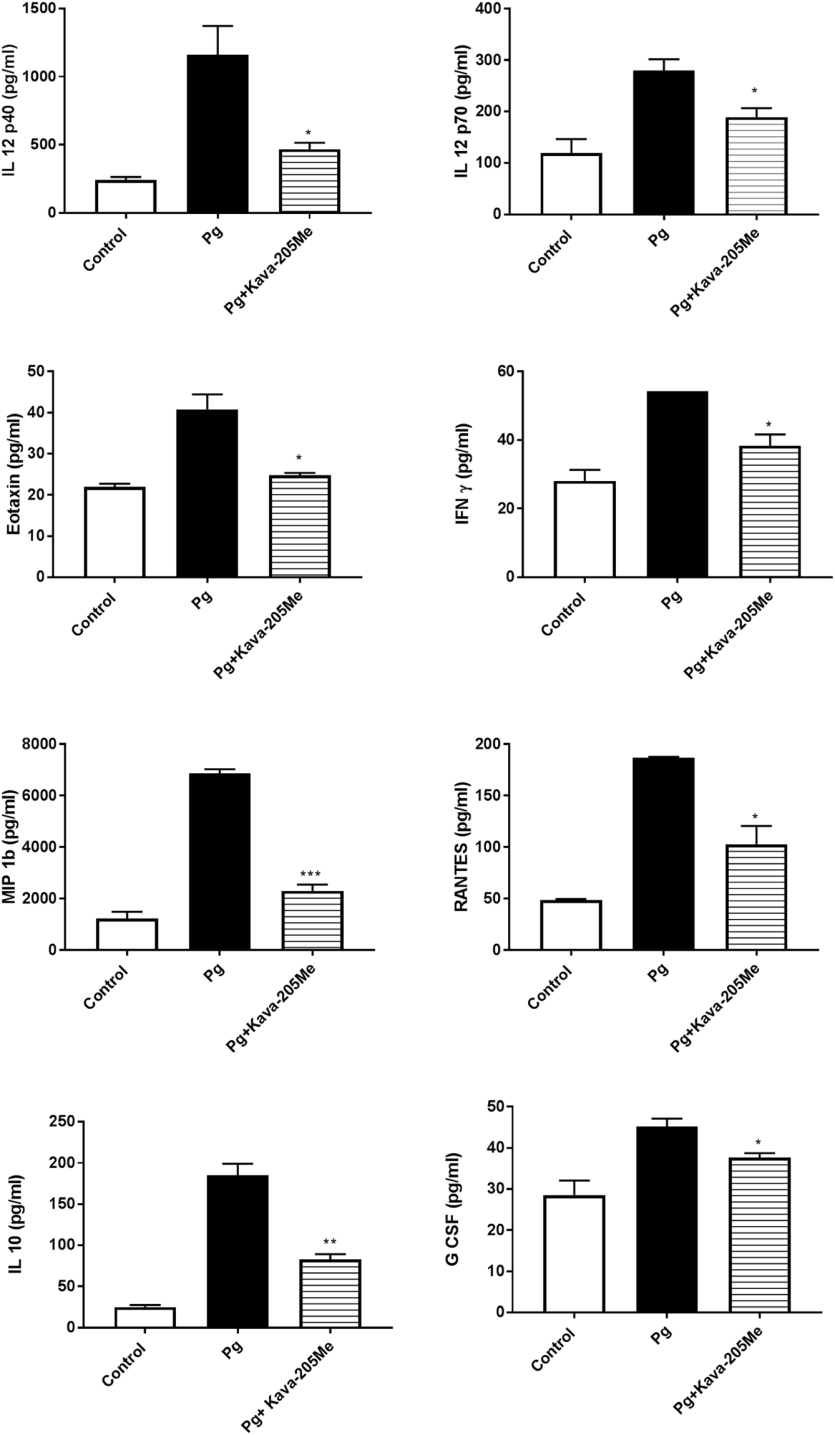
Effect of Kava-205Me treatment on cytokine secretion from *P. gingivalis*-stimulated BMM. The BioPlex Pro Mouse Cytokine 23-plex Assay was utilized to determine the cytokine levels in BMM culture supernatants stimulated with *P. gingivalis* for 4 hrs. The data presented are means of 3 replicates. Eight of 23 cytokines showed significant reductions after Kava-205Me treatment (50 μg/ml). Unpaired t-test was performed for *P. gingivalis* vs *P. gingivalis* + Kava-205Me (*p < 0.05; **p < 0.01; ***p < 0.001).

*P. gingivalis* is able to induce sustained recruitment of immune cells such as neutrophils and T-lymphocytes, whose presence is associated with excessive inflammation and tissue destruction at infectious sites. To evaluate the effect of Kava-205Me on the secretion of chemokines and cytokines from BMM exposed to *P. gingivalis*, a multiplex analysis was performed that compared *P. gingivalis* infected cells with and without treatment with Kava-205Me. As expected, *P. gingivalis* infection significantly increased secretion of IL-12, IFN- γ, MIP-1β, RANTES, IL-10, G-CSF and eotaxin. Treatment with Kava-205Me (50 μg/ml) significantly reduced the concentrations of such chemokines and cytokines (Fig. 3). Moreover, the gene expression of ACP5, an osteoclast specific gene, was evaluated in BMM infected with *P. gingivalis* with or without Kava-205Me treatment. As observed for inflammatory cytokines, *P. gingivalis* increased significantly ACP5 gene expression and Kava-205Me treatment reduced its expression illustrating a potential protective effect on bone (Fig. 2C).

### Kavain-205Me reduced *P. gingivalis*-induced calvarial destruction

**Figure 4:**
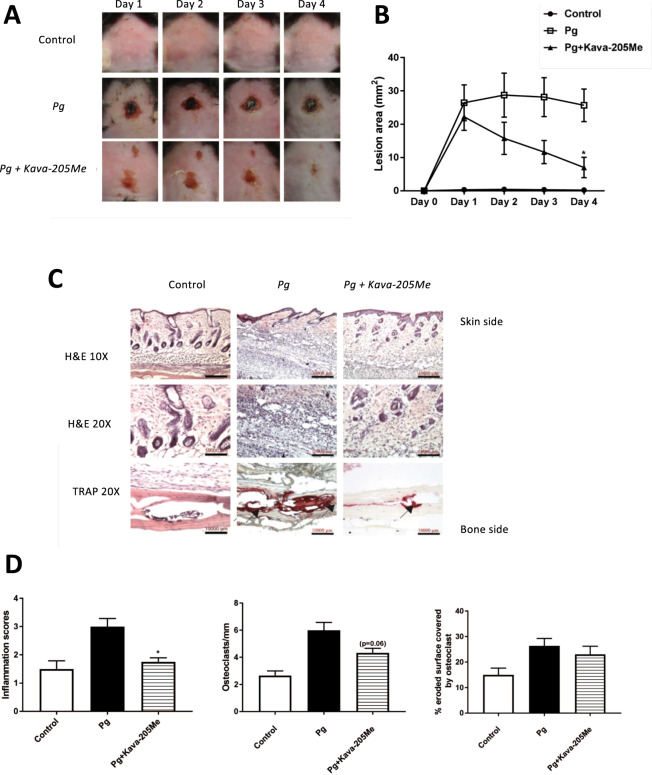
Effect of Kava-205Me on calvarial healing. Injection of Kava-205Me (1 mg) significantly decreased *P. gingivalis*-induced calvarial destruction. After 4 days, calvarial wounds in treated mice had largely closed while wounds in untreated mice still exhibited large lesions **(A**,**B)**. At the histological level, a decreased number of inflammatory cells within soft tissue was observed in the Kava-205Me group, and fewer for TRAP positive cells were observed **(C)**. Quantitative evaluation of histological changes following Kava-205Me treatment (inflammation score; osteoclast index, osteoclast-covered surface) **(D)**. *p < 0.05.

In an acute model of *P. gingivalis* infection, Kava-205Me was administered to evaluate its anti-inflammatory and pro-healing properties *in vivo*. In this calvarial model, injection of *P. gingivalis* induced formation of a cutaneous abscess characterized by dermal inflammation and bone destruction (Fig. 4). Administration of Kava-205Me significantly increased the rate of healing (Fig. 4B). At the histological level, treatment with Kava-205Me reduced significantly soft tissue inflammation (Fig. 4C,D). Moreover, a trend of reduction in osteoclastic activity was also observed after treatment (Fig. 4D).

### Systemic administration of Kava-205Me significantly reduced infective arthritis- associated joint destruction

**Figure 5:**
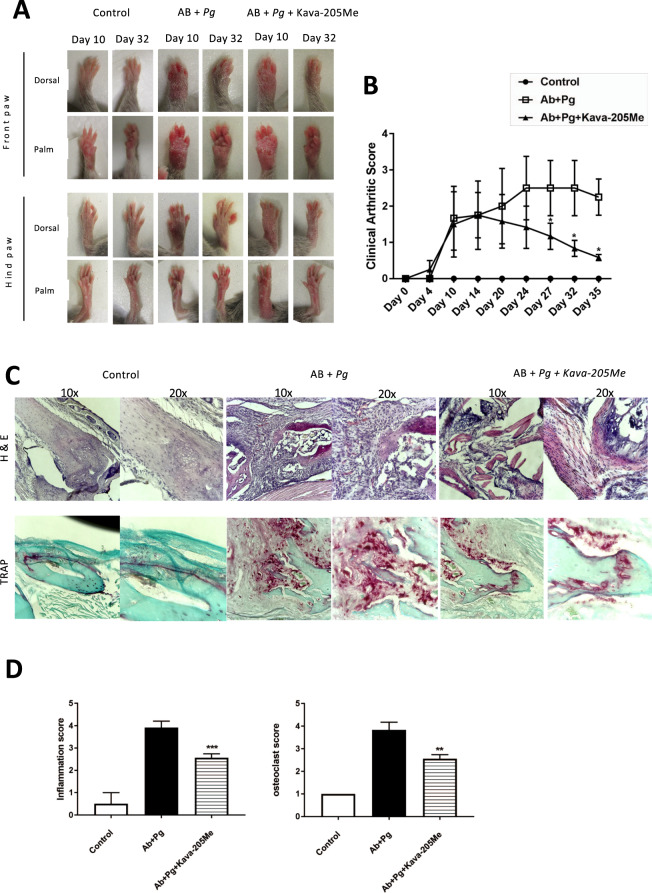
Effect of Kava-205Me in infective arthritis model. Clinical examination of forepaws (**A**) and clinical score of arthritis **(B)** were evaluated daily in all groups. Pictures were taken at 10 and 32 days. Note the swollen paws in *P. gingivalis* + AB group and the significant reduction in Kava-205Me treated group; *p < 0.05. Histological views of the joint **(C)** (H-E staining for inflammation and TRAP staining. Histological sections performed at the joint site are representative of each group. AB + *P. gingivalis* injected group was associated with intense infiltrate of inflammatory cells, predominantly neutrophils macrophages and lymphocytes. Furthermore, signs of edema and synovial hyperplasia were clearly observed. Treatment with Kava-205Me reduced significantly such signs of inflammation and tissue destruction. Quantitative evaluation of histological changes following Kava-205Me treatment (inflammation score; osteoclast score) **(D)**. *p < 0.05.

To evaluate the systemic effect of Kava-205Me, an infective arthritis was induced in mice by the combined insults of *P. gingivalis* and AB peritoneal injection. After 10 days, significant paw swelling was observed, confirming the establishment of joint inflammation and a destructive process (Fig. 5). Interestingly, systemic administration of Kava-205Me significantly decreased *P. gingivalis*-induced paw swelling at the 32 day time point (Fig. 5A,B). This anti-inflammatory effect was confirmed histologically (Fig. 5C). A significant reduction of the inflammatory infiltrate in paw tissue sections was observed in animals exposed to *P. gingivalis* and treated by Kava-205Me. In contrast, untreated *P. gingivalis* exposed animals. exhibited a significant inflammatory infiltrate dominated by neutrophils and lymphocytes at the same time point (Fig. 5C). Congruent with these results, Kava-205Me treatment of *P. gingivalis*-exposed animals was also found to reduce the osteoclastic activity of the infiltrate, as evidenced by a reduction in the number of TRAP positive multinucleated cells in contact with bone. In contrast, *P. gingivalis*-exposed animals left untreated exhibited a significant increase of the number of TRAP positive multinucleated cells in contact with bone (Fig. 5C,D).

## Discussion

This study identifies a promising new kavain derivative, Kava-205Me, with clear anti-inflammatory and pro-healing properties *in vitro* and *in vivo*. Kava-205Me demonstrated the ability of reducing inflammatory cytokines and chemokines secretion in human and murine macrophages induced by *P. gingivalis* infection. *In vivo*, it significantly reduced *P. gingivalis* -associated inflammation and tissue destruction in both acute and chronic infection models.

Kavain is a natural product, long used as an anti-anxiety and anti-inflammatory drug in traditional medicines of the Pacific islands. It has been shown to reduce secretion of pro-inflammatory cytokines, especially TNF-α, in several cell types^12–14,23^. However, due to the side effects (hepatic, neurologic, and dermatological toxicity) associated with its use and to optimize its pharmacological properties, there is a recognized need to identify new kavain analogs^24^. Kava-205Me is a newly identified active compound bearing a para-methyl substituted phenyl ring which varies electronically and sterically from the para-chloro analog, where the chlorine atom is electron withdrawing, decreasing the chemical and metabolic stability of the vinylogous anhydride linkage. When compared to natural kavain, which is susceptible to degradation because of its lactone functional group, Kava-205Me may be metabolically stabilized by the replacement of the lactone function with a cyclic α,β-unsaturated enone system, (or vinylogous anhydride) thereby showing enhanced anti-inflammatory effects compared to kavain. These unique, but subtle structural features of Kava-205Me may be responsible for its enhanced performance as shown in this study. We speculate that methylation of Kava-205Me most likely increases efficacy by enhancing metabolic stability.

Herein, the anti-TNF-α property of Kava-205Me was demonstrated *in vitro* in macrophages of both human and murine origin. Additionally, it was shown that Kava-205Me was also able to modulate the concentrations of secreted cytokines and chemokines including IL-12, IFN-γ and RANTES in cultures of *P. gingivalis* infected macrophages. Also, an impact was observed at short term on IL-10, a cytokine well-known to be increased in the first phase of *P. gingivalis* infection to escape immune response suggesting an impact of Kava-205Me on the rescue of immune response^10,25^. These cytokines are associated to periodontitis development and have already been shown to be activated by *P. gingivalis* infection, contributing to inflammation and neutrophil recruitment at lesion sites^26–29^. These cytokines and others are key mediators involved in the host-immune response and tissue destruction through activation of proteases such as matrix metalloproteinases (MMPs)^27^. Nevertheless, we also demonstrated, for the first time, the protective role of Kava-205Me on bone destruction. Treatment with Kava-205Me reduced significantly ACP5 expression in infected BMM, a marker of osteoclast differentiation, activation and proliferation. Such effect was already demonstrated for others drugs such as fluvastatin^30^ emphasizing the potential for Kava-205Me in the management of bone destructive diseases.

The anti-inflammatory properties of kavain and its derivatives in multiple cell types such as macrophages, osteoblasts and even adipocytes are now well described^12,14,15,18^. Interestingly, such effects are mediated by several pathways involved in NFκB activation including ERK/LITAF^13^ and peroxisome proliferation–activated receptor γ coactivator α (PGC-1α)^12^ leading to the control of other detrimental biological events such as oxidative stress. This broad spectrum of action will be of interest in the management of several acute and chronic inflammatory diseases of infectious origin. However, it is clear that not every analog behaves like its parent molecule and therefore, a large-scale analysis should be conducted to determine all molecular pathways affected by Kava-205Me.

To confirm the observed effect *in vivo*, administration of Kava-205Me was administered to mice in acute and chronic inflammation models. In a model of *P. gingivalis* induced calvarial destruction, injection of Kava-205Me at the lesion site promoted wound healing. *P. gingivalis* is a destructive bacterium and its inoculation at calvarial site induces formation of an abscess, inflammation of soft tissues, and destruction of underlying bone^10^. Treatment with Kava-205Me significantly reduced recruitment of inflammatory cells and subsequent bone destruction. Such effects may be correlated to the observed reduction of secretion of pro-inflammatory cytokines; however, additional studies are required to determine which phase(s) of the inflammatory response^31^ is/are most affected by Kava-205Me. Kava-205Me also contributed to reduce joint inflammation and tissue destruction in an infective arthritis model triggered by *P. gingivalis*. Treatment reduced inflammatory cell infiltration within soft tissues and also decreased osteoclastic activity. These results are consistent with those obtained in a model of ovariectomy-induced bone loss where kavain was suggested as a candidate drug for the pharmacological management of osteolytic diseases^15^. Indeed, it has been demonstrated that kavain is able to inhibit receptor activator of nuclear factor‐κB ligand (RANKL)-induced osteoclast differentiation and associated bone resorption^15^.

The anti-inflammatory properties of Kava-205Me led to decreases in *P. gingivalis*-induced inflammation and bone destruction. This promising compound should be studied further for its potential therapeutic applications in other acute and chronic inflammatory diseases and development of adapted scaffold should be conducted.

### Supplementary information

Dataset
