# Author Correction: Identification of a Kavain Analog with Efficient Anti-inflammatory Effects

**DOI:** 10.1038/s41598-024-63102-y

**Published:** 2024-05-31

**Authors:** Olivier Huck, Xiaxian Han, Hannah Mulhall, Iryna Gumenchuk, Bin Cai, James Panek, Radha Iyer, Salomon Amar

**Affiliations:** 1 Faculté de Chirurgie-Dentaire, Université de Strasbourg, 8 rue Sainte-Elisabeth, 67000 Strasbourg, France; 2 INSERM (French National Institute of Health and Medical Research), UMR 1260, Regenerative Nanomedicine, Fédération de Médecine Translationnelle de Strasbourg (FMTS), Strasbourg, France; 3 Departments of Pharmacology, Microbiology and Immunology, New York Medical College, Valhalla, NY 10595 USA; 4 Department of Chemistry, Boston University, Boston, MA USA

Correction to: *Scientific Reports* 10.1038/s41598-019-49383-8, published online 10 September 2019

The original version of this Article contains errors. Due to errors during figure assembly, in Figure 5C the panel “TRAP” AB + Pg + Kava-205Me, 10 × shows an image that belongs to the dataset “TRAP” AB + Pg, 10 × and therefore partially overlaps with the corresponding panel.

The corrected Figure 5 and accompanying legend appear below as Figure 1.

**Figure 1:**
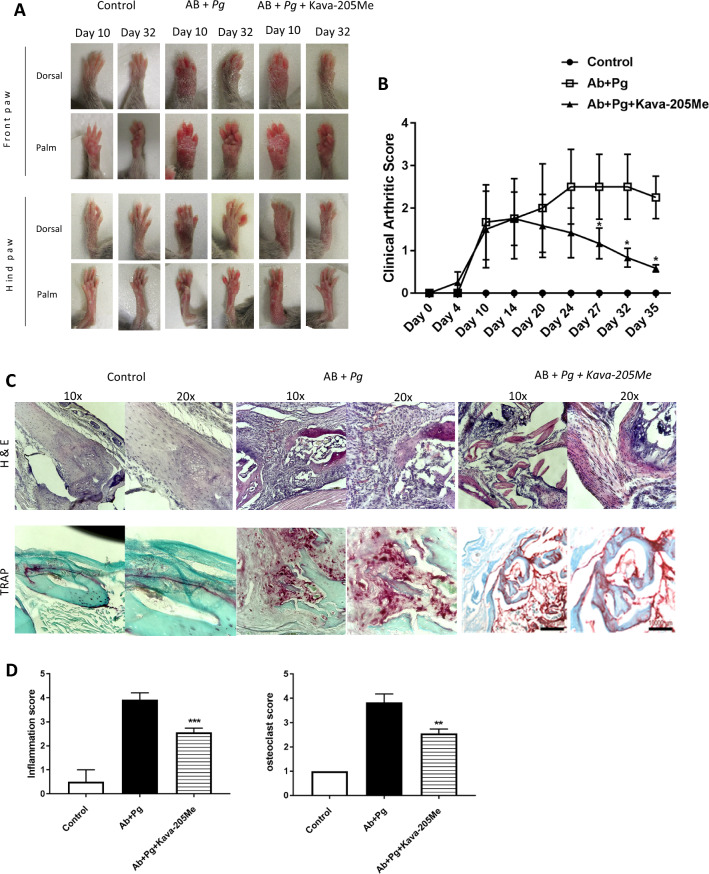
Effect of Kava-205Me in infective arthritis model. Clinical examination of forepaws (**A**) and clinical score of arthritis (**B**) were evaluated daily in all groups. Pictures were taken at 10 and 32 days. Note the swollen paws in *P. gingivalis* + AB group and the significant reduction in Kava-205Me treated group; *p < 0.05. Histological views of the joint (**C**) (H-E staining for inflammation and TRAP staining. Histological sections performed at the joint site are representative of each group. AB + *P. gingivalis* injected group was associated with intense infiltrate of inflammatory cells, predominantly neutrophils macrophages and lymphocytes. Furthermore, signs of edema and synovial hyperplasia were clearly observed. Treatment with Kava-205Me reduced significantly such signs of inflammation and tissue destruction. Quantitative evaluation of histological changes following Kava-205Me treatment (inflammation score; osteoclast score) (**D**). *p < 0.05.

In addition, there is an error in the subsection ‘Mouse calvarial bone resorption model’ of the Materials and Methods section. The sentence.

“The 8- to 12-week-old wild-type C57BL/6 J mice used in this study were purchased from Taconic Laboratories (Germantown, NY).”

should read:

“The 8- to 22-week-old wild-type C57BL/6 J mice used in this study were purchased from Taconic Laboratories (Germantown, NY).”

