# Supplementary material for: Identification of a Kavain Analog with Efficient Anti-inflammatory Effects
Source: Sci Rep. 2019 Sep 10;9:12940. doi: 10.1038/s41598-019-49383-8 (PMC6737110; doi:10.1038/s41598-019-49383-8)
Supplement: Supplementary file 1 — Dataset [file 41598_2019_49383_MOESM1_ESM.docx]

***Identification of a Kavain Analog with Efficient Anti-inflammatory Effects***

***Olivier Huck, Xiaxian Han, Hannah Mulhall, Iryna Gumenchuk, Bin Cai, James Panek, Radha Iyer, Salomon Amar***

***Chemical structure of Kavain analogs***

***Supplementary Figure S1: Chemical structure of tested Kavain analogs.*** Figure S1 provides a selected subset of the most active compounds.

***Evaluation of Kava-205Me cytotoxicity***

Macrophages (RAW 264.7 (TIB-71, ATCC, Manassas, VA, USA)) were cultured in RPMI 1640 medium (Life Technologies, NY, NY, USA) with 10% FBS at 37° in 5% CO2 atmosphere. Cells were then stimulated by *P.gingivalis* and/or treated with Kava-205Me (10 and 50 μg/ml) for 24h. A trypan blue exclusion test of cell viability was conducted to determine the percentage of viable cells as described previously by Strober et al.^1^


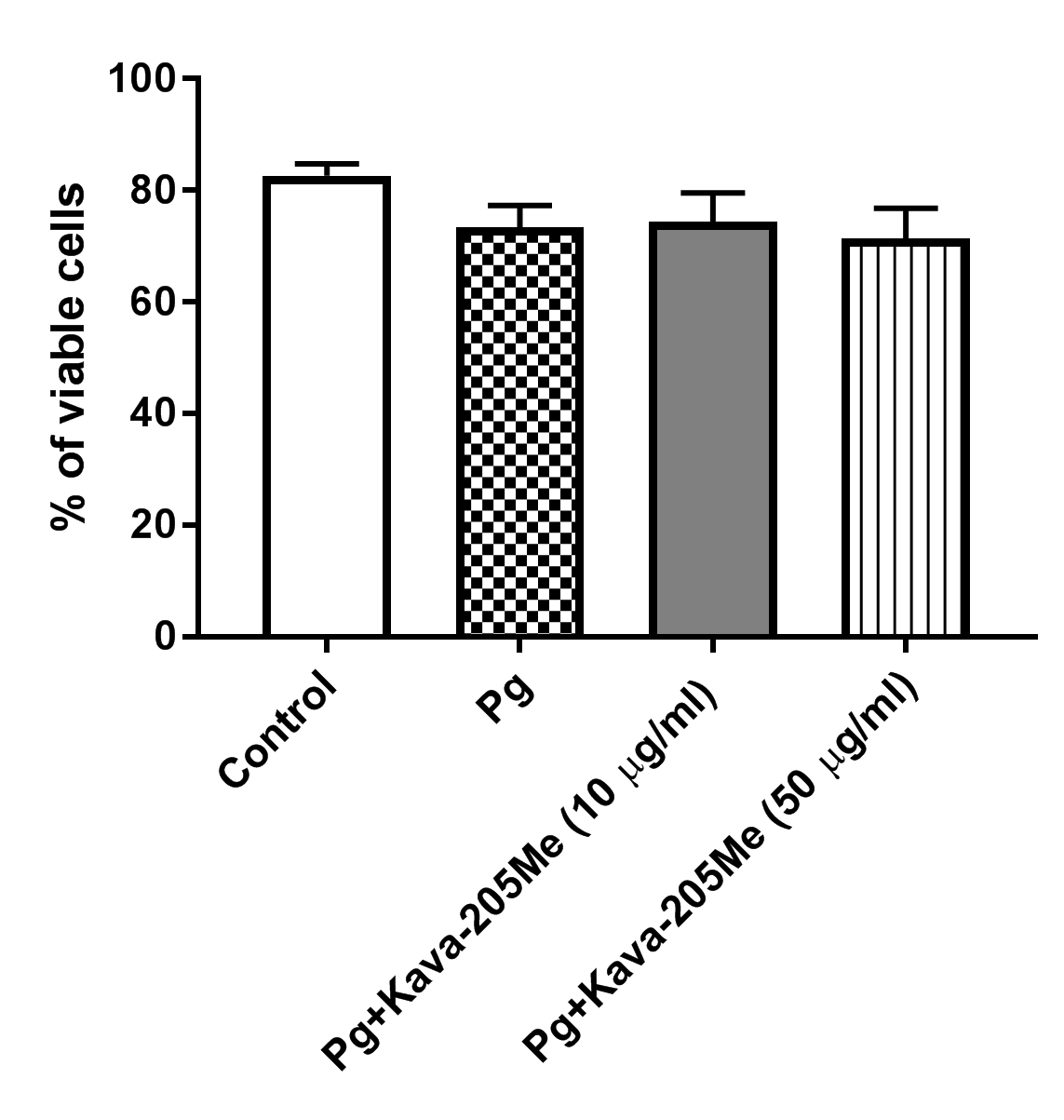
After 24h of treatment, no significant cytotoxicity was observed for the two tested concentrations of Kava-205Me (Figure 2).

***Supplementary Figure S2: Evaluation of Kava-205Me cytotoxicity.*** RAW cells were infected with *P.gingivalis* (*Pg*) (MOI:20) and treated for 24h with Kava-205Me (10 and 50 μg/ml). Results are presented as mean percentage of viable cells +/- SD. Experiment has been performed at least in triplicate.

***Reference***

1. Strober W. Trypan blue exclusion test of cell viability. *Curr Protoc Immunol.* **21**(3B) (2001). doi: 10.1002/0471142735.ima03bs21.
